# Editorial: Metabolism-based omics integrations, biosensors, and molecular mechanisms in human cancers

**DOI:** 10.3389/fonc.2023.1159545

**Published:** 2023-03-16

**Authors:** Yi-Fang Yang, Kuo-Wang Tsai, Peter Mu-Hsin Chang, Yu-Chan Chang

**Affiliations:** ^1^ Department of Medical Education and Research, Kaohsiung Veterans General Hospital, Kaohsiung, Taiwan; ^2^ Department of Research, Taipei Tzu Chi Hospital, Buddhist Tzu Chi Medical Foundation, New Taipei City, Taiwan; ^3^ Department of Oncology, Taipei Veterans General Hospital, Taipei, Taiwan; ^4^ Faculty of Medicine, National Yang Ming Chiao Tung University, Taipei, Taiwan; ^5^ Institute of Biopharmaceutical Sciences, National Yang Ming Chiao Tung University, Taipei, Taiwan; ^6^ Department of Biomedical Imagine and Radiological Sciences, National Yang Ming Chiao Tung University, Taipei, Taiwan

**Keywords:** metabolism, omics, tumor microenvironment, fatty acid, cancer

Metabolism is one of the most important aspects of carcinogenesis. During the cancerous stage, metabolism changes rapidly to match the corresponding phenotype (1). And while there are various therapeutic strategies such as chemotherapy, radiotherapy, and immunotherapy in cancer treatment, most of the options are accompanied by unsatisfactory drug efficacy, inactive signaling transductions, and relative resistance (2, 3). Tumor heterogeneity and the tumor microenvironment have been considered the main reasons behind these consequences, but metabolism is also accompanied by the causal transition and reprogramming described above (4–6).

Admittedly, metabolism is constantly changing and difficult to quantify, and using omics concepts to unravel the mystery is an efficient and straightforward approach. Key canonical pathways or responses to target compound therapy can be achieved through the integration of multidisciplinary omics profiles (7, 8).

Metabolism can be roughly divided into the biosynthesis of monosaccharides, fatty acids, amino acids, nucleotides, etc. Monosaccharides have been studied the most widely, and their derived inhibitors and tracers are also widely used (9). Discussion of fatty acids has been a hot topic in recent years. In addition to their involvement in cell membrane composition, fatty acid oxidation is also involved in various cancer phenotypes, oxidative stress, and mitochondrial function (10). Zhou et al. described some aberrant mRNAs and proteins expressed in colorectal cancer (CRC). They integrated multiple datasets, then selected EPHX2 to act as the core gene under their algorithm and interpretation. They determined that the loss of EPHX2 function was increased ROS and lipid droplets in CRC cells. In their manipulation, EPHX2 also appeared to affect the proportion of immune cells. Melero-Fernandez de Mera et al. identified metabolic signatures between glioblastoma stem-like cells and exosomes. They noticed changes in a number of fatty acid products, especially ceramides. They revealed the abundance and structural composition of ceramide. Regarding the ratio of C16/C24:1 Cer, different events and subsequent strategies can be clearly identified. Li et al. reported that dehydroepiandrosterone (DHEA), which is a steroid hormone molecule in the lipid environment, is based on its anti-cancer and lipolytic effects. DHEA acts synergistically with irinotecan *via* WNT signaling to inhibit cancer stemness in head and neck cancer.

The comprehensive decipherment of metabolic events and the interplay of multiple omic layers are the main goals. Jo et al. recruited and analyzed the available literature and selected Secretogranin V as a diagnostic biomarker for pancreatic adenocarcinoma. They relied on machine learning modeling and pipelines to evaluate the SCG5 gene significantly associated with survival and various clinicopathological factors. Du et al. combined pharmacokinetic and metabolomic characterization in animal models. They used Sotorasib, a specific KRAS G12C inhibitor, and then established pharmacokinetic and pharmacometabolomic profiles for further analysis. Nineteen metabolic pathways were disrupted following sotorasib treatment, most notably taurine and hypotaurine metabolism, as determined by high-performance liquid chromatography-tandem mass spectrometry (HPLC-MS/MS). These studies on biomarkers predictive of drug treatment contribute to the diagnosis of patients with genetic alterations. For sure, some detection tools still have limitations and weaknesses. Therefore, He et al. illustrated the comparison and advantages among spatial metabolomics platforms. Mass spectrometric imaging based on matrix-assisted laser desorption ionization (MALDI), desorption electrospray ionization (DESI), and air flow-assisted desorption electrospray ionization (AFADESI) are discussed in turn and compared with each other. They provide detailed evidence for finding key metabolites in a variety of cancers using the approaches described above, including breast cancer, esophageal cancer, lung cancer, and glioblastoma.

These findings will serve as a niche for basic and translational research in cancer metabolism. While systemic changes in metabolism cannot be fully deciphered, they can be deeply explored for specific populations and events. With these targets, derived biosensors useful for imaging, quantification, and tracking will make great contributions. Detailed molecular mechanisms will be applied in drug development and regulation of metabolic events. Most importantly, the concept of metabolism-based omics integration should be applied to more cancer research.

## Author contributions

All authors listed have made a substantial, direct, and intellectual contribution to the work and approved it for publication.
